# Supplementary figures and images for: ZJUSAH Classification: A New Classification for Primary Brainstem Hemorrhage
Source: Life (Basel). 2023 Mar 21;13(3):846. doi: 10.3390/life13030846 (PMC10059730; doi:10.3390/life13030846)

Type 2

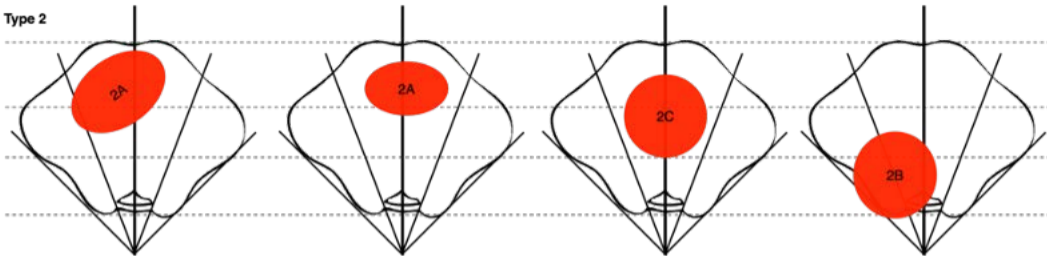

Type 3

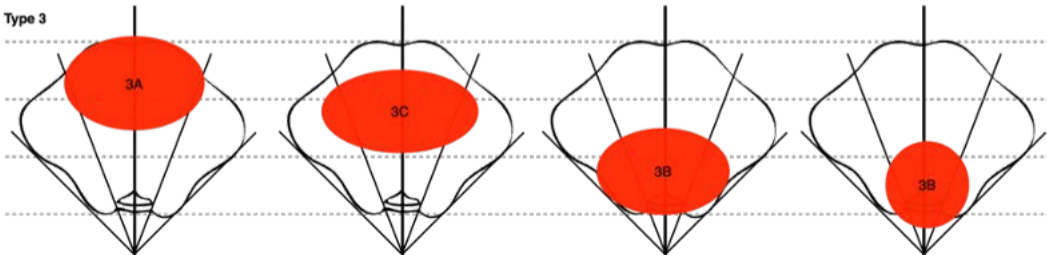

Supplement: Supplementary file 1 [file life-13-00846-s001.zip › life-2137298-supplementary.pdf]
